# Mobile Phone Ownership Is Not a Serious Barrier to Participation in Studies: Descriptive Study

**DOI:** 10.2196/mhealth.8123

**Published:** 2018-02-19

**Authors:** Emily J Harvey, Leslie F Rubin, Sabrina L Smiley, Yitong Zhou, Hoda Elmasry, Jennifer L Pearson

**Affiliations:** ^1^ Truth Initiative Schroeder Institute for Tobacco Research and Policy Studies Washington, DC United States; ^2^ Department of Psychology American University Washington, DC United States; ^3^ Department of Health, Behavior, and Society Bloomberg School of Public Health Johns Hopkins University Baltimore, MD United States

**Keywords:** smoking, smartphone ownership, online survey screener, ecological momentary assessment, tobacco products/utilization, electronic cigarettes, observational study, United States

## Abstract

**Background:**

Rather than providing participants with study-specific data collection devices, their personal mobile phones are increasingly being used as a means for collecting geolocation and ecological momentary assessment (EMA) data in public health research.

**Objective:**

The purpose of this study was to (1) describe the sociodemographic characteristics of respondents to an online survey screener assessing eligibility to participate in a mixed methods study collecting geolocation and EMA data via the participants’ personal mobile phones, and (2) examine how eligibility criteria requiring mobile phone ownership and an unlimited text messaging plan affected participant inclusion.

**Methods:**

Adult (≥18 years) daily smokers were recruited via public advertisements, free weekly newspapers, printed flyers, and word of mouth. An online survey screener was used as the initial method of determining eligibility for study participation. The survey screened for twenty-eight inclusion criteria grouped into three categories, which included (1) cell phone use, (2) tobacco use, and (3) additional criteria

**Results:**

A total of 1003 individuals completed the online screener. Respondents were predominantly African American (605/1003, 60.3%) (60.4%), male (514/1003, 51.3%), and had a median age of 35 years (IQR 26-50). Nearly 50% (496/1003, 49.5%) were unemployed. Most smoked menthol cigarettes (699/1003, 69.7%), and had a median smoking history of 11 years (IQR 5-21). The majority owned a mobile phone (739/1003, 73.7%), could install apps (86.8%), used their mobile phone daily (89.5%), and had an unlimited text messaging plan (871/1003, 86.8%). Of those who completed the online screener, 302 were eligible to participate in the study; 163 were eligible after rescreening, and 117 were enrolled in the study. Compared to employed individuals, a significantly greater proportion of those who were unemployed were ineligible for the study based on mobile phone inclusion criteria (*P*<.001); yet, 46.4% (333/717) of the individuals who were unemployed met all mobile phone inclusion criteria.

**Conclusions:**

Inclusion criteria requiring participants to use their personal mobile phones for data collection was not a major barrier to study participation for most respondents who completed the online screener, including those who were unemployed.

**Trial Registration:**

ClinicalTrials.gov NCT02261363; https://clinicaltrials.gov/ct2/show/NCT02261363 (Archived by WebCite at http://www.webcitation.org/6wOmDluSt)

## Introduction

Ecological momentary assessment (EMA), a real-time data capture method, has been increasingly used as a viable method to collect data from research participants over time and in various contexts outside of a research lab[1-3]. EMA focuses on collecting data and recording subject experiences at a particular moment in the context of the research participants’ natural environment [4]. The types of devices used in EMA data collection have changed with the development of, access to, and utilization of new technology [5]. Whereas EMA once entailed that the participants keep written records, most real-time data is now collected via handheld electronic devices, such as tablets and cell phones[5-7]. Along with EMA, geotracking is another method that is being used more frequently to collect information regarding the participants’ real-time physical location or surroundings (eg, Global Positioning System [GPS] coordinates, home, office) by obtaining GPS data from their mobile phones or other GPS-enabled devices. Geotracking also allows the identification and analysis of those locations in relation to ones’ actions and/or experiences [8-10]. The technology used for both EMA and geotracking within public health research has developed alongside advancements in consumer electronics, and the methods can now be implemented through apps on the study participants’ personal mobile phones [5,8].

The use of mobile phones has become a method for EMA and geotracking data collection over the past ten years [7,11,12]. Such a preference may be attributed to the surge in mobile phone ownership among the general population. Between 2011 and 2016, there was a 42% increase in mobile phone ownership, and 77% Americans now own a mobile phone [13]. The unique features of mobile phones include internet accessibility and the ability to download and install apps.

Instead of providing participants with study-specific handheld electronic devices, personal mobile phones are increasingly being used as a means for collecting EMA and geotracking data, providing a noncoercive (eg, no free phone for study participation), low-cost tool to collect data in real-time [1]. While the majority of US adults own a mobile phone, it cannot be assumed that a potential study participant will have access to a mobile phone [13]. Factors such as socioeconomic status (SES), which encompasses employment, income, and educational levels, may impact mobile phone ownership. Researchers must consider the potential participants’ mobile phone ownership and usage when conducting studies utilizing EMA and geotracking methods. Thus, understanding a potential participant’s access to a mobile phone is important for interpreting the generalizability of results when implementing these methods. The purpose of this study was to (1) describe the sociodemographic characteristics of respondents to an online survey screener assessing their eligibility to participate in the “Moment Study”: a mixed methods study that examined e-cigarette initiation among adult cigarette smokers using geolocation and text message-based EMA data collected via the participants’ personal mobile phones, and (2) examine how eligibility criteria requiring mobile phone ownership and an unlimited texting plan affected study eligibility.

## Methods

### Study Design

Data come from a parent study called the “Moment Study”, a mixed method longitudinal study that examined factors influencing e-cigarette initiation using a convenience sample of adult daily smokers residing in Washington, DC [14]. Briefly, the parent study involved data collected over three-weeks, and included: 1) geotracking; 2) EMA; 3) individual interviews; 4) biosamples; and 5) an online follow-up survey 30-days after the participant’s last study visit. Ethics approval for the study was obtained from the Chesapeake IRB (Pro00008526). Text message-based EMA data were collected via mobile phones. A secure, automated text message-based EMA system prompted participants to respond to 6 random text message surveys a day for 21 days. Participants also initiated text message surveys whenever they smoked a cigarette or used an e-cigarette. Geotracking data was collected via an app downloaded to participants’ mobile phones. The app collected one tracking point every five minutes [14]. The analyses presented here include data from the online survey screener only.

### Recruitment and Eligibility

We recruited a convenience sample of adult smokers via public advertisements, free weekly newspapers, printed flyers, and word of mouth. The advertisements included a link to an online survey, which was the initial screening tool. The online survey screened for twenty-eight inclusion criteria grouped into three categories, which included (1) mobile phone use, (2) tobacco use, and (3) additional inclusion criteria.

The inclusion criteria for mobile phone use derived from the technological needs for EMA and geotracking data collection. We required daily mobile phone use and a preexisting unlimited text messaging plan to ensure that participants were comfortable sending and receiving texts and the number of text messages sent and received over the course of three weeks did not pose a financial burden on the participants. We also required the participants to have an Android or iPhone mobile phone because the geotracking app was only available on these operating systems. Device requirements were not expected to be restrictive, as Android and iPhone are the leading mobile phone operating systems in the United States with Android holding 56.4% and iPhone accounting for 42% of the market as of January 2017 [15].

In addition to the mobile phone inclusion criteria, we also required that participants be adult (≥18 years) daily cigarette smokers with restricted past 30-day use of other tobacco products. Additional inclusion criteria included age, pregnancy status, and both physical and mental health status (Table 2). Prior to study enrollment, participants deemed eligible by the online screener were re-screened via telephone by study personnel to confirm that they met the inclusion criteria.

### Statistical Analyses

Descriptive statistics were used to summarize the sociodemographic characteristics of individuals who completed the online screener, overall and eligibility by virtue of mobile phone ownership, and to describe the frequency of respondents meeting the study criteria. Observations with missing data were listwise deleted. Chi square tests and Mood’s median tests (a special case of chi square tests) were used to test the equality of proportions or medians. Statistical significance was set to a *P*-value of 0.05. All analyses were conducted using Stata 14.2 (Stata Corp, College Station, TX, USA).

## Results

A total of 1003 individuals took the online screener. Most respondents were African American (605/1003, 60.3%), male (514/1003, 51.3%), and had a median age of 35 years (IQR 26-50). Nearly all the respondents lived in Washington, DC, Virginia or Maryland (976/1003, 97.3%), and approximately half (496/1003, 49.5%) were unemployed. Most smoked menthol cigarettes (699/1003, 69.7%) and had smoked for a median of 11 years (IQR 5-21) (Table 1).

Of the 1003 respondents to the online screener, 28.5% (286/1003) were ineligible because of mobile phone inclusion criteria. Differences by sociodemographic characteristics and mobile phone inclusion criteria were evident. African Americans (57.4% vs 70.5%, *P*<.001) and menthol smokers (72.0% vs 68.8%, *P*<.001) made up greater proportions of people who were ineligible owing to mobile phone inclusion criteria. Additionally, people who were ineligible owing to mobile phone inclusion criteria were older (median age of 47 vs 32, *P*<.001) and had smoked for more years (median years smoked being 20 vs 10, *P*<.001). Of those who met the mobile phone inclusion criteria, 53.6% (384/717) were employed; conversely, among those who did not meet mobile phone criteria, 68.9% (197/286) were unemployed.

Among the other inclusion criteria, 44.0% of the unemployed individuals met all tobacco use inclusion criteria, and 93.0% of unemployed individuals met all the additional inclusion criteria; there was no difference in eligibility by employment status for the tobacco use or all other inclusion criteria (results not shown).

**Table 1:** Characteristics of individuals who took the initial online screener, overall and by mobile phone inclusion criteria, in the Washington, DC area.

Characteristic		Mobile phone inclusion criteria			
		Overall (N=1003)	Met inclusion criteria (>N=717)	Did not meet inclusion criteria (N=286)	*P* value
**Gender, n (%)^a^**					<.001
	Female	478 (47.7)	346 (48.3)	132 (46.2)	
	Male	514 (51.3)	371 (51.7)	143 (50.0)	
**Race, n (%)^a^**					<.001
	White	248 (24.7)	193 (27.0)	55 (20.0)	
	African American	605 (60.3)	411 (57.4)	194 (70.5)	
	Asian	39 (3.9)	35 (4.9)	4 (1.5)	
	Native Hawaiian	1 (0.1)	1 (0.1)	0 (0)	
	American Indian	2 (0.2)	1 (0.1)	1 (0.4)	
	Other	52 (5.2))	41 (5.7)	11 (4.0)	
	More than 1 race	44 (4.4)	34 (4.7)	11 (3.6)	
Hispanic, n (%)		77 (7.7)	58 (8.1)	19 (6.6)	.530
Age, median (IQR^b^)^c^		35 (26-50)	32 (25-45)	47 (32-55)	<.001
Years smoking, median (IQR)^c^		11 (5-21)	10 (6-20)	20 (11-35)	<.001
Menthol smoker n (%)		699 (69.7)	493 (68.8)	206 (72.0)	<.001
Live in the DC metro area, n (%)		976 (97.3)	708 (98.9)	268 (93.7)	.201
**Employment, n (%)^a^**					
	Employed	473 (47.2)	384 (53.6)	89 (31.3)	<.001
	Not employed	496 (49.5)	333 (46.4)	197 (68.9)	

^a^The following variables had missing data: gender (1%), race (1.2%), employment (3.3%).

^b^IQR: interquartile range.

^c^Mood’s median tests were used to test for differences in median age and years smoked.

**Table 2:** Percentage of screened individuals who satisfied mobile phone, tobacco use, or additional inclusion criteria in the online screener (N=1003).

Inclusion criteria			n (%)
**Mobile phone criteria**			
	**Have Android or iPhone**		739 (73.7)
		Mobile phone allows app installation	871 (86.8)
		Use mobile phone daily	898 (89.5)
		Unlimited text message plan	871 (86.8)
		Meet all the mobile phone inclusion criteria	717 (71.5)
**Tobacco use criteria**			
	**Daily smoker**		890 (88.7)
		≥5 years of smoking	772 (77.0)
		≥8 cigarettes a day	689 (68.7)
		Use of little cigars no more than 5 times in the last 30 days	814 (81.2)
		Use of cigars no more than 5 times in last the 30 days	998 (99.5)
		Use of hookah no more than 5 times in the last 30 days	996 (96.3)
		No use of pipe (with tobacco, not including hookah) in the last 30 days	995 (95.2)
		No use of chewing tobacco in the last 30 days	995 (99.2)
		No use of dip/snuff in the last 30 days	997 (99.4)
		No use of snus in the last 30 days	1000 (99.7)
		No use of nicotine products (like gum, patches) in the last 30 days	961 (95.8)
		No use of e-cigarettes in the last 30 days	833 (83.1)
		Interested in trying an e-cigarette	689 (68.7)
		Meet all tobacco use inclusion criteria	426 (42.5)
**Additional inclusion criteria**			
	Not on cessation medication		947 (94.4)
	Not breastfeeding or planning to become pregnant		975 (97.2)
	No heart disease/uncontrolled blood pressure		941 (93.8)
	No psychosis		943 (94.0)
	No suicidal thoughts		949 (94.6)
	Not enrolled currently in an alcohol treatment program		940 (93.7)
	Not out of town for more than 5 nights in the next 6 weeks		933 (93.0)
	18 years or older		1002 (99.9)
	Reside in the DC metro area		976 (97.3)
	Willing to travel to data collection site four times in three weeks		949 (94.6)
	Meet all additional inclusion criteria		916 (91.3)

The majority of the respondents owned an iPhone or Android mobile phone (739/1003, 73.7%) and could install apps on their mobile phone (871/1003, 86.8%). Many used their mobile phones daily (898/1003, 89.5%) and had an unlimited text messaging plan (871/1003, 86.8%). Most (717/1003, 71.5%) met the mobile phone inclusion criteria, and 91.3% met all the additional inclusion criteria; however, only 42.5% (426/1003) met all the tobacco inclusion criteria. Overall, 302 individuals were eligible to participate in the study with 163 eligible after being rescreened; 117 were ultimately enrolled in the study (Table 2).

## Discussion

### Principal Considerations

This study describes the sociodemographic characteristics of individuals responding to an online screener, and examines how eligibility criteria requiring mobile phone ownership affected study eligibility. A total of 1003 individuals completed the online screener of whom 73.7% (739/1003) owned a mobile phone; of the mobile phone owners, 86.8% (871/1003) could install apps, 89.5% (898/1003) used their mobile phones daily, and 86.8% (871/1003) had an unlimited text messaging plan. While our mobile phone inclusion criteria were strict, 46.4% (333/717) of the unemployed respondents still met the criteria, suggesting that mobile phone ownership is not a major barrier to study participation, even for unemployed individuals.

These findings also have broader relevance for the tobacco control field, which increasingly uses mobile phone technology to examine tobacco product use, surveil tobacco retail environments, and deliver smoking cessation interventions [10,16,17]. Smoking is concentrated among low-SES groups with sometimes unstable employment; provision of a free mobile phone as part of study participation may be coercive for members of such vulnerable groups. Examination of who has access to a mobile phone is essential to assessing how planning a protocol using methods such as EMA or geotracking might affect ethical considerations, study recruitment, and generalizability of the findings.

### Limitations

The study utilized an online screener, which may have been a barrier to individuals who lacked internet access. Had we initially screened potential participants via telephone, we may have found that a greater proportion of respondents were ineligible because of lack of mobile phone ownership. Additionally, given that this study was conducted in Washington, DC, our findings may not be generalizable to other settings. Moreover, it is important to consider that all data collected in this study were self-reported responses to the online screener questions. Therefore, the respondents’ mobile phone and tobacco use may have been under- or over-reported owing to social desirability and recall bias.

### Conclusions

This research suggests that using the participants’ own mobile phones for data collection, including geotracking and text messaging, was not a barrier to study participation for the majority of the respondents who took the initial online screener. Employment was related to fulfilling the mobile phone inclusion criteria; however, nearly half of the eligible respondents were unemployed. As mobile phone ownership continues to grow, researchers should consider using the participants’ own mobile phones as feasible data collection devices given their study’s target population.

## Abbreviations

EMA: ecological momentary assessment
GPS: Global Positioning System
IQR: interquartile range
